# Building capacity: a cross-sectional evaluation of the US Training Institute for Dissemination and Implementation Research in Health

**DOI:** 10.1186/s13012-019-0947-6

**Published:** 2019-11-21

**Authors:** Cynthia A. Vinson, Mindy Clyne, Nina Cardoza, Karen M. Emmons

**Affiliations:** 1 National Cancer Institute, 9609 Medical Center Drive, Rockville, MD 20850 USA; 2 Kaiser Foundation Research Institute, PO Box 8040, Redwood City, CA 94063 USA; 3 Harvard T.H. Chan School of Public Health, 677 Huntington Avenue, Boston, MA 02115 USA

**Keywords:** Training, Dissemination, Implementation

## Abstract

**Background:**

In 2011, the National Institute of Health (NIH) initiated the Training in Dissemination and Implementation Research in Health (TIDIRH) program. Over its first 5 years, TIDIRH provided an in-person, week-long training to 197 investigators who were new to the dissemination and implementation (D&I) field. This paper evaluates the long-term impact of TIDIRH on trainees’ use of D&I methods, collaborations, and research funding.

**Methods:**

Trainees were selected to participate through a competitive process. We compared the 197 trainees to 125 unselected applicants (UAs) whose application score was within one standard deviation of the mean for all trainees’ scores for the same application year. A portfolio analysis examined electronic applications for NIH peer-reviewed funding submitted by trainees and UAs between 2011 and 2019. A survey of trainees and UAs was conducted in 2016, as was a faculty survey among the 87 individuals who served as TIDIRH instructors.

**Results:**

A major goal of TIDIRH was to build the field, at least in part through networking and collaboration. Thirty-eight percent of trainees indicated they had extensive contact with faculty following the training, and an additional 38% indicated they had at least limited contact. Twenty-four percent of trainees had extensive collaboration with other fellows post-TIDIRH, and 43% had at least limited contact. Collaborative activities included the full range of academic activities, including manuscript development, grant writing, and consultation/collaboration on research studies.

The portfolio analysis combining grant mechanisms showed that overall, TIDIRH trainees submitted more peer-reviewed NIH grants per person than UA and had significantly better funding outcomes (25% vs 19% funded, respectively). The greatest difference was for large research project, program/center, and cooperative agreement grants mechanisms.

**Conclusions:**

Overall, this evaluation found that TIDIRH is achieving its three primary goals: (1) building a pipeline of D&I investigators, (2) creating a network of scholars to build the field, and (3) improving funding outcomes for D&I grants.

Contributions to the literature

- As the field of dissemination and implementation (D&I) research continues to grow, formalized training programs have been implemented to train investigators. However, there is limited information on the impact of these training programs in helping investigators in becoming successful D&I researchers.
- This study outlines unique methods used for conducting an outcome evaluation of the Training Institute for Dissemination and Implementation Research in Health.
- The outcomes from the evaluation highlight how this targeted training program helps build the field and increases productivity related to D&I research.

## Background

The National Institutes of Health (NIH) began earnestly focusing on dissemination and implementation (D&I) research in 2001 when new D&I programs started at the National Cancer Institute (NCI) and the National Institute of Mental Health (NIMH) [1]. A key goal was to stimulate more research funding focused on the integration of evidence-based programs within clinical and community settings. In 2005, nine NIH institutes, centers, and offices (ICOs) collaborated on the first trans-NIH program announcement focused on dissemination and implementation research in health [2] which is open to both domestic and international grantees. The announcement has been reissued five times and is now supported by 20 of the 27 ICOs at NIH [3].

When the trans-NIH funding opportunity for D&I opened in 2005, few investigators had sufficient expertise in D&I research. To help investigators develop successful D&I grant applications responsive to the funding opportunity, the NCI and the NIMH held a technical assistance workshop in 2005. When the NIH began hosting the Annual Conference on the Science of Dissemination and Implementation in Health in 2007, this workshop was incorporated at the end of the meeting; since 2014 the meeting has been hosted by Academy Health.

In recognition of the need to further develop the field of dissemination and implementation science, in 2011, NIH initiated the Training Institute in Dissemination and Implementation Research for Health (TIDIRH) program. Over its first 5 years, TIDIRH provided an in-person, week-long training to 197 investigators who were new to the D&I field. When TIDIRH began, there were few, if any, formal training programs or resources for methodological training and education in D&I research.

TIDIRH had three primary goals: (1) build a pipeline of investigators who had strong D&I research competencies and provide resources to train others at their institutions, thus expanding the impact of TIDIRH beyond those in attendance; (2) create a network of scholars to build the field; and (3) improve funding outcomes for D&I grants [4]. A small core faculty planned the training each year, with additional faculty added as presenters. The trainings were held at institutions across the country that had a strong corpus of work in D&I research and a dedicated faculty member available to serve as the chair. TIDIRH provided a combination of didactic and interactive presentations, and large and small group work that focused on the development of individual investigator-initiated (R01 level) D&I grant applications. Table 1 provides an overview of the course content over the 5 years.

**Table 1:**
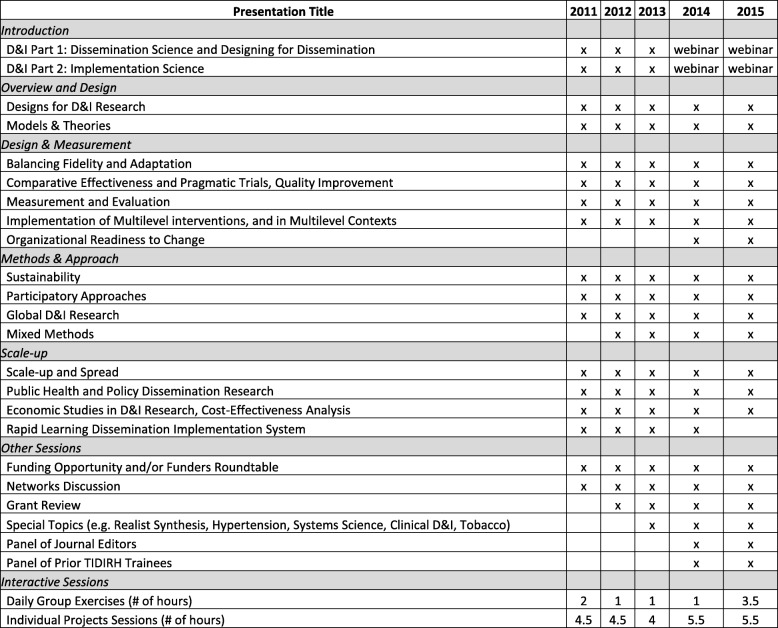
TIDIRH course content (2011–2015)

This paper builds on the initial evaluation of TIDIRH from 2011 [4] and evaluates the longer-term impact of TIDIRH on trainees’ use of D&I methods, collaborations, and receipt of research funding. We posed four key questions related to the training’s contributions including the following: (1) was the training effective at increasing trainees’ use of D&I methods?; (2) did trainees have more engagement in scientific leadership/activities focused on D&I?; (3) did TIDIRH foster new collaborations; and (4) did trainees fare better with peer-reviewed D&I grant applications than unselected applicants?

## Methods

The evaluation was multipronged and included objective analysis of both peer-reviewed funding opportunities and conference presentations, as well as analysis of survey responses completed by (1) individuals participating in the training (“trainees”), (2) a subset of highly competitive applicants not selected for the training (“unselected applicants”), and (3) faculty. Unselected applicants (UA) were identified as those whose application score was within one standard deviation of the mean for all trainee scores for the same application year. Nine individuals who were unselected upon their initial application but applied and were admitted in a subsequent year were included in the evaluation as trainees for the year they were accepted and removed from the pool the year(s) they were not selected.

A *content analysis* examined electronic applications submitted for TIDIRH by trainees and UA between 2011 and 2015. Gender (male or female), academic ranking (professor, associate professor, assistant professor, or other), and location (domestic or international) of each applicant were identified through their application.

In the fall of 2016, a *survey of trainees* and UA was conducted to assess more about individual experiences related to D&I research. Three follow-up e-mails were sent to non-respondents between August and September 2016. The response rate among trainees was 50% and UA was lower at 22%.

A *faculty survey* was sent to all 87 individuals who participated as instructors at least once in TIDIRH between 2011 and 2015. All surveys were conducted between August 3, 2016, and September 30, 2016, and the response rate among faculty was 49%.

An analysis was conducted in May 2019 to evaluate the utilization of D&I research methods from a non-self-reported perspective. This was achieved by comparing trainees and UA who were presenters at the Annual Conference on the Science of Dissemination and Implementation in Health from 2011 through 2018. Presentations at the conference were chosen as an objective source since the selection of presenters is done through a blinded, peer-review process. Presentation information was ascertained through archived records from each annual conference.

A *portfolio analysis* was conducted in February 2019 using PI and Co-PI name searches through an internal NIH grants database, Query, View Report (QVR). The analysis was performed on peer-reviewed grants with project start dates that followed the subsequent review cycle after completion of TIDIRH training relative to each individual’s year of application, up through project start dates of February 9, 2019. The abstract, specific aims, and research strategy of grant submissions were reviewed to determine whether the grant focused on D&I research. Submissions were excluded if D&I research was not involved.

To determine whether trainees and UAs were similar in gender and rank, and for analysis of survey results, a chi-square test was performed with *p* < .05 statistical significance. For portfolio analysis and D&I conference presentations evaluation, we compared the percentage of trainees to the percentage of UA using *t* test with *p* < .05 as statistical significance. These data analyses were performed using SAS/STAT® software [5].

## Results

Between 2011 and 2015, there were 1100 applicants to TIDIRH, and 199 were selected to participate through a competitive process (Table 2). Two selected individuals were unable to attend, resulting in 197 trainees. Trainees were compared with 125 UA. Trainees and UA were similar with respect to gender distribution ((75% and 76% female, respectively). Academic rank and distribution of trainee’s geographic location (e.g., domestic or international) was also similar between trainees and UA (Table 3).

**Table 2:** Applications received for TIDIRH

Year	Total applications received =	# accepted	Accepted (%)	# not accepted	Not accepted (%)
2011	264	36	13.6	228	86.4
2012	204	36	17.6	168	82.4
2013	162	45	27.8	117	72.2
2014	289	41	14.2	248	85.8
2015	181	41	22.7	140	77.3
Total	1100	199	18.1	901	81.9

**Table 3:** Demographics

	Trainees *n* = 197 (%)	UA *n* = 125 (%)
Gender		
Male	49 (25)	30 (24)
Female	148 (75)	95 (76)
Rank		
Assistant	60 (30)	33 (26)
Associate	25 (13)	24 (19)
Professor	14 (7)	12 (10)
Other	98 (50)	56 (45)
Location		
Domestic	174 (88)	113 (90)
International	23 (12)	12 (10)

### Was the training effective in development and use of D&I methods?

We queried the extent to which trainees utilized the D&I research methods received at TIDIRH for purposes other than peer-review grant applications, which was a focus of the training. Survey findings indicated that the methods were being well utilized for a range of activities, including quality and performance improvement studies (73%), manuscripts (92%), and presentations (95%).

Presentations at the Annual Conference on the Science of Dissemination and Implementation in Health, currently hosted by Academy Health, were analyzed for the percentage of trainees and UA for all training years combined. A higher percentage of trainees were presenters (30%) compared to UA (18%) (*p* ≤ 0.01). A higher percentage of trainees did oral (45%, *p* ≤ 0.01) and panel (14%, *p* ≤ 0.01) presentations compared to UA (30% and 4%); the percentage of poster presentations were higher for UA (66% vs 41%, *p* = 0.02).

We queried about training still needed to develop a successful career in D&I research (Table 4). Report of additional training needs was similar among trainees and UA except for having opportunities for interaction with other scholars learning D&I research methods and having intensive training. These data suggest that UAs were not able to replicate these types of experiences elsewhere.

**Table 4:** Respondents’ perception of additional D&I research training needs

Additional D&I research training needs	Trainees (%)	UA (%)
Follow-up Lectures	52	46
Informal discussion sessions	45	43
Local mentoring	33	25
Interaction with other trainees	30	64
Intensive trainings	34	71

We queried faculty for their perceptions of the effectiveness of TIDIRH as a vehicle for training the next generation of D&I researchers. On a 5-point scale, from “not at all effective” to “very effective,” the average rating was 4.03; 78% of faculty respondents felt that TIDIRH has been very effective or extremely effective. On a 5-point scale, from “not at all” to “significantly,” 92% of faculty felt that, since the time of their participation in TIDIRH, the focus on D&I research has increased nationally at least “modestly”; 52% felt that it has increased “significantly.” On a 5-point scale, from “not at all” to a “large extent,” 55% of respondents felt that this change could be attributed to TIDIRH to a “moderate” or “large extent.” Some faculty with participation in a single year indicated that they gave neutral ratings because they did not have a full picture of the training impact, and thus the summary scores may be slightly suppressed as a result.

### Did trainees have more engagement in scientific leadership/activities focused on D&I?

Nineteen percent of trainees and 3.6% of UA represented D&I research expertise as a reviewer on an NIH study section (standing or ad hoc member, *p* = 0.04), and 11% of trainees and 0% of UA served on a non-NIH study section related to D&I, although this difference was not significant. About twice as many trainees served as manuscript reviewers related to D&I (47% vs 25%, respectively, *p* = 0.04). Editorial board participation and engagement in scientific activities unrelated to D&I was similar among both groups.

### Did participation in TIDIRH result in new collaborations?

We queried trainees as well as faculty about collaborations developed at TIDIRH. Thirty-eight percent of trainees indicated that they had extensive contact with faculty following the training, and an additional 38% indicated that they had at least limited contact. Over 50% of the faculty reported that they developed collaborations with trainees. Collaborative activities included the full range of academic activities, including manuscript development, grant writing, and consultation/collaboration on research studies.

There was also an indication of collaboration development among fellows. Twenty-four percent of trainee respondents had extensive collaboration with other fellows post-TIDIRH, and 43% had at least limited contact. TIDIRH also appeared to stimulate new collaborations among faculty, with 40% reporting that they developed new collaborations with another faculty.

### Did participation in TIDIRH lead to better funding outcomes?

The portfolio analysis combining grant mechanisms shows that overall, TIDIRH trainees submitted more NIH grants per person than UA, although not statistically significant (*p* = 0.10). Trainees had better outcomes, with 25% of submitted NIH grants funded, compared with 19% among UA (*p* = 0.04). The difference between groups was particularly pronounced for larger grants (R01, R18, P20, U01, U19, and UH2) [6], with 23% of trainees and 15% of UA who submitted these awards receiving funding (*p* = 0.03). The award rate for smaller grants (R03, R21, R31) [6] was similar among trainees (24%) and UA (26%). There were no significant differences in submissions or award for NIH career development awards.

## Discussion

Overall, this evaluation found that TIDIRH is achieving the three primary goals it started with (1) building a pipeline of D&I investigators, (2) creating a network of scholars to build the field and (3) improving funding outcomes for peer-reviewed D&I research grants. The faculty experts who participated in TIDIRH rate it as very important and effective. In response to an open-field question about the single most important outcome from TIDIRH, faculty responses clustered into four thematic areas: (1) field and capacity building, via creating a pipeline of trained researchers; (2) networking and building a community of practice; (3) ensuring that the field has scientific rigor and key D&I research competencies; and (4) increasing the success of D&I grant applications.

TIDIRH was effective at building a pipeline of investigators who integrated D&I research methods into their research. That trainees were well-distributed across rank also suggests that such trainings have benefit across career levels. There was a high use of the methods gained in TIDIRH and a high-level of train-the-trainer type activities that occurred, among both trainees and faculty, achieving a program goal to extend the impact of TIDIRH beyond the training trainees.

There was a high-level of engagement among trainees and faculty that led to improved collaboration and building of effective D&I research networks. This type of networking is something that UA report not being able to get elsewhere. Networking for trainees continues to occur at the annual D&I conference and other national meetings.

TIDIRH trainees were more productive and successful in terms of D&I research compared to UA. The impact on awards was particularly strong for a large research project, program/center, and cooperative agreement grants. This suggests that TIDIRH succeeded in stimulating more NIH-funded research in D&I.

Strengths of this study include having a reasonable comparison group by including UAs who were essentially next in line for participation if there had been more slots available. This was possible because of the popularity of the TIDIRH program relative to the small number of slots available. Survey data was supplemented by an NIH peer-reviewed portfolio analysis evaluating objective outcomes related to the impact of TIDIRH on NIH-funded research. Objective analysis regarding participation in peer-reviewed presentations at the annual D&I conference also supplemented the self-reported survey results on the utilization of methods. The portfolio and conference participation analyses were available for all trainees and applicants, irrespective of their participation in the survey.

### Limitations

Limitations that should be noted include that both surveys went out in August which may have limited responses and may have contributed to the low response rate for UA which limits insight into the current level of engagement in D&I research for these investigators. Additionally, since the portfolio analysis was restricted to NIH grants, results do not reflect grant submissions funded through other sources. Similarly, D&I research presentations could have been given at other conferences. However, since the annual D&I conference is the premier conference for D&I research, it is expected that this is a good measure for skill utilization.

Our evaluation was restricted to training years 2011–2015. In 2016, TIDIRH shifted from a week-long immersive training program to a three-month “hybrid” training course which includes a 3-month interactive on-line training course followed by a 2-day in-person training. Many of the features of the original training were maintained to ensure engagement, and opportunities for collaboration were available to trainees, including time for trainees to interact in large groups as well as facilitated small-group discussions both online and in person. Materials from the online training course are available on-line from NCI [7]. The new format for the training is currently being evaluated.

## Conclusion

This study demonstrates the potency of targeted training programs as a field-building activity that can lead to higher productivity and collaborations over a relatively short period of time. In 2019, TIDIRH continues to be a popular training program, with more than 200 applicants applying for the 50 available training slots. With an eye toward sustainability, the training has been packaged for delivery in different settings. In 2018, NCI launched the Training Institute for Dissemination and Implementation Research in Cancer (TIDIRC) which uses customized content from TIDIRH to train approximately 50 cancer researchers annually. NCI also collaborated with University College Cork, Ireland, so they could adapt and deliver TIDIRH-Ireland. Discussions with additional partners are underway regarding adaptation and delivery in other countries and settings.

## Data Availability

The grant application and funding dataset developed during the study can be replicated in NIH Reporter database and the dataset for the D&I annual conference presentations is available from the corresponding author based on reasonable request.

## Abbreviations

D&I: Dissemination and implementation
ICO: Institutes, centers, and offices
NCI: National Cancer Institute
NIH: National Institutes of Health
NIMH: National Institute of Mental Health
TIDIRC: Training Institute for Dissemination and Implementation Research in Cancer
TIDIRH: Training Institute for Dissemination and Implementation Research in Health
UA: Unaccepted applicants
